# Structural Advances in Respiratory Syncytial Virus: Implications for Vaccine and Antiviral Development

**DOI:** 10.3390/microorganisms14051130

**Published:** 2026-05-16

**Authors:** Xuanwei Huang, Caner Akıl, Peijun Zhang

**Affiliations:** 1 Chinese Academy of Medical Sciences Oxford Institute, University of Oxford, Oxford OX3 7BN, UK; xuanwei.huang@lmh.ox.ac.uk; 2 Division of Structural Biology, Nuffield Department of Medicine, University of Oxford, Oxford OX3 7BN, UK; 3 Diamond Light Source, Harwell Science and Innovation Campus, Didcot OX11 0DE, UK

**Keywords:** respiratory syncytial virus, membrane fusion, antiviral strategies, structure-based vaccine design, antibodies, cryo-ET, cryo-EM

## Abstract

Respiratory syncytial virus (RSV) remains a leading cause of severe lower respiratory tract disease in infants, older adults, and immunocompromised individuals. Over the past decade, advances in structural biology, particularly cryo-electron microscopy (cryo-EM) and cryo-electron tomography (cryo-ET), have transformed our understanding of RSV architecture, dynamics, and the mechanisms of entry and replication. High-resolution structures of the prefusion F glycoprotein (pre-F) and its complexes with neutralizing antibodies established the rationale for structure-guided antigen stabilization and directly enabled the development of the first licensed RSV vaccines. Complementary structures of the ribonucleoprotein, polymerase complex, and matrix lattice have broadened therapeutic targets beyond F. Here, we summarize these structural advances; review current structure-guided vaccine, antibody, and antiviral development efforts; and highlight priorities for next-generation vaccines and therapeutics.

## 1. Introduction

Over the past decade, advances in cryo-electron microscopy (cryo-EM) and cryo-electron tomography (cryo-ET) have profoundly expanded our understanding of viral architecture and dynamics [1,2]. These technologies have revealed structures of enveloped viruses such as influenza [3], SARS-CoV-2 [4,5], HIV-1 [6], respiratory syncytial virus (RSV), and several other structurally characterized viruses. In particular, cryo-ET has enabled the observation of viruses in near-native states, capturing transient events, from membrane fusion [3,7,8], transcription regulation [9,10], and capsid [11,12] and nucleocapsid [4,13] assembly to budding [14,15]. Together, these advances demonstrate that these approaches not only deepen our molecular understanding of viruses but also provide the foundation for structure-guided vaccine [16,17] and antiviral development, exemplified by the breakthroughs achieved in respiratory syncytial virus (RSV) research [18,19] and by the development of the HIV-1 capsid inhibitor Lenacapavir, a structure-guided antiviral drug now approved for clinical use [20,21].

The RSV genome consists of a single-stranded, negative-sense RNA molecule that encodes 11 distinct viral proteins [22]. Based on differences in the G protein sequence, RSV can be classified into two major antigenic subtypes, RSV-A and RSV-B [23]. Since its discovery in 1955 [24], RSV has remained one of the leading causes of virus-associated infant mortality worldwide. RSV is responsible for approximately 33 million infections and more than 100,000 deaths in children under five years old annually [25]. The significant global disease burden imposed by RSV has therefore made the development of effective vaccines and antiviral therapies a major public health priority.

In recent years, increased structural insight into RSV has enabled the development of rationally designed vaccines and therapeutics. Detailed studies of RSV architecture [26], particularly the prefusion structure of its fusion (F) glycoprotein [27,28], have formed the basis for the first generation of structure-based RSV vaccines. These include the stabilized prefusion F (pre-F) constructs now utilized in three licensed vaccines: Arexvy (GSK), Abrysvo (Pfizer), and mRESVIA (Moderna) [18]. Beyond the F protein, structural studies have also advanced our understanding of other essential viral components, including the ribonucleoprotein (RNP) complex by X-ray crystallography [29], the RdRp by cryo-EM [30], and the organization of the matrix (M) lattice by cryo-ET [31], each playing crucial roles in transcription, replication, and virion assembly.

This review focuses on recent advances in the structural and mechanistic understanding of RSV. Here, we summarize key findings that clarify the organization and function of major viral proteins and examine how these insights inform current vaccine development efforts. Finally, we discuss remaining challenges and future directions relevant to next-generation RSV vaccines and antiviral therapeutics. The literature and regulatory developments discussed in this review were assessed up to early March 2026, and regulatory status is reported primarily for the United States, unless otherwise specified.

## 2. RSV Genome and Virion Architecture

### 2.1. Genome

**Figure 1:**
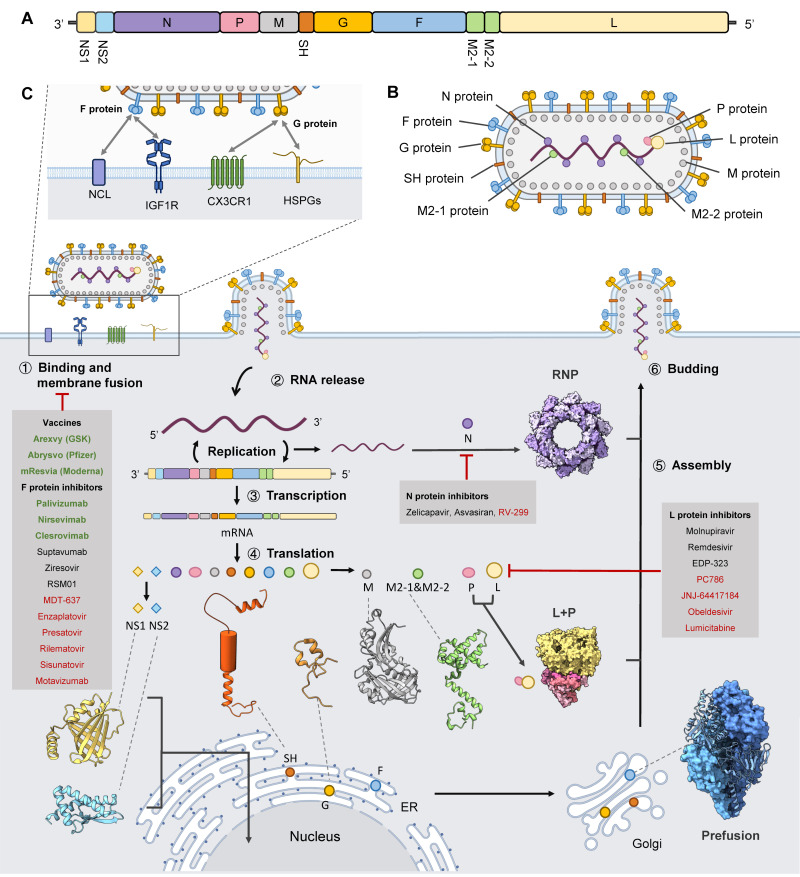
RSV genome, life cycle, and structures of RSV components. (**A**) Schematic representation of the RSV genome organization. RSV carries a negative-sense RNA genome in which ten genes encode eleven viral proteins. NS1, non-structural protein 1; NS2, non-structural protein 2; N, nucleocapsid protein; P, phosphoprotein; M, matrix protein; SH, small hydrophobic protein; G, attachment protein; F, fusion protein; M2-1, transcription antitermination factor; M2-2, replication regulatory protein; L, large polymerase protein. (**B**) Filamentous virion architecture of RSV. (**C**) RSV life cycle and sites targeted by antiviral interventions. The G and F proteins mediate viral attachment and membrane fusion, enabling entry of the viral genome into host cells, followed by transcription and translation. Representative three-dimensional structures of RSV-encoded proteins are shown. NS1, PDB 5VJ2; NS2, PDB 7LDK; N, PDB 2WJ8; P, PDB 6PZK; M, PDB 4V23; SH, PDB 2NB7 and 2NB8; G, PDB 6BLH; F, PDB 4JHW; M2–1, PDB 4C3D; and L, PDB 6PZK. The F protein is shown in its prefusion trimeric conformation (PDB: 4JHW). The L–P polymerase complex is also displayed (PDB: 6PZK). The non-structural proteins NS1 and NS2 enter the nucleus to regulate host antiviral immune responses, primarily by suppressing interferon transcription and signalling. The SH, G, and F proteins are assembled in the endoplasmic reticulum and transported through the Golgi apparatus to the cell surface, while the N protein encapsidates the viral RNA in the conformation shown (PDB: 2WJ8). Together with other viral proteins, these components assemble into progeny virions that bud from the host cell membrane. The grey boxes in the figure indicate therapeutic strategies targeting each viral protein, including vaccines and inhibitors. Green text denotes approved interventions, black text indicates candidates under clinical trials, and red text represents programs that have been discontinued.

The RSV genome is nonsegmented, approximately 15.2 kb in length, belonging to the Pneumoviridae family and the Orthopneumovirus genus [22,32]. It encodes 11 proteins arranged sequentially from the 3′ to 5′ end in the order listed in the glossary table (Table 1) (Figure 1A). This linear organization is flanked by leader (Le) and trailer (Tr) regions that regulate RNA synthesis and genome encapsidation [33]. The F, G, and SH proteins reside on the viral membrane, whereas the N, P, L, and M2 proteins are positioned inside the envelop, with M residing underneath the viral envelope (Figure 1B) [34].

The first two genes, NS1 and NS2, encode non-structural proteins that antagonize host innate immunity by suppressing interferon signalling [22]. NS1 regulates the host interferon response by preventing the recruitment of transcriptional regulators [35,36]. NS2 induces autophagy by modulating the stability of Beclin-1. In addition, it inhibits the production of inflammatory cytokines and suppresses apoptosis, thereby impairing key antiviral immune defences [37]. N protein encapsulates the viral RNA, forming the RNP complex that serves as the template for transcription and replication [29].

SH protein, G protein, and F protein are all transmembrane glycoproteins. Among them, the F and G proteins are essential for viral entry into host cells [19]. Because the F protein is highly conserved among different RSV strains, it serves as a major target for vaccine development [38]. The SH protein functions as a viroporin that increases membrane permeability and modulates host cell apoptosis [39]. In addition to these membrane-associated proteins, several internal viral proteins coordinate RNA synthesis and virion assembly. The P protein functions as an essential polymerase cofactor, bridging N and L to stabilize the polymerase [30,32]. The L protein is a multifunctional enzyme containing distinct catalytic domains responsible for RNA synthesis, capping, and methylation [18]. These proteins form the core machinery required for RSV transcription and replication. M protein is located beneath the viral envelope and is essential for RSV assembly [40]. Consistent with coordinated regulation of viral gene expression, the M2 gene encodes two regulatory proteins, M2-1 and M2-2, that play distinct but complementary roles in organizing RSV RNA synthesis [41,42]. Together, the organization of the RSV genome and its gene-specific functions ensures precise regulation of transcription, replication, and assembly.

### 2.2. Structures of RSV Components

The RSV virion is a filamentous, enveloped particle approximately 130 nm in diameter. The viral envelope is underlain by a M protein layer that organizes the filamentous morphology of the virus, with a surface layer enriched in glycoproteins and an internal RNP complex that forms the core of the virion (Figure 1B) [18]. Advances in structural biology have enabled detailed characterization of these viral components and revealed how their molecular architectures support the viral life cycle. Cryo-ET studies confirm that RSV virions can appear spherical or filamentous, although filamentous particles predominate in infection [26]. The RNP complex is observed within the virion interior, frequently running along the M2-1 layer beneath the matrix lattice [31]. The RSV envelope contains two transmembrane glycoproteins (G and F) and the small hydrophobic protein SH (Figure 1B), which play central roles in viral attachment and entry. The G protein is heavily glycosylated and functions primarily as an attachment factor that interacts with host receptors to mediate viral proximity to the cell membrane [43]. At present, no high-resolution structure of the full-length RSV G protein has been determined because it is largely intrinsically disordered, with a structurally defined central conserved domain (CCD). The crystal structure of the CCD revealed a compact fold exposing the Cysteine–X–X–X–Cysteine (CX3C) motif, providing a structural basis for receptor interaction and antibody recognition [44]. Following G protein’s mediation, the F protein acts as the primary fusion factor and undergoes a dramatic conformational transition to drive membrane fusion [45]. McLellan et al. were the first to determine the prefusion structure of the RSV F protein [27]. They stabilized the pre-F conformation and preserved antigenic site Ø, a key prefusion-specific neutralizing epitope [28]. The SH protein is implicated in modulating host cell signalling and viral pathogenicity. Structural insight into SH was provided by solution NMR studies demonstrating that SH assembles into a pentameric ion channel within lipid membranes [46].

Inside virions, the N protein encapsulates the negative-sense genomic RNA to form the RNP complex, which both protects the genome and serves as the template for transcription and replication (Figure 1C). A crystallographic nucleocapsid-like N–RNA assembly showed that each N monomer binds roughly seven nucleotides and organizes into a helical array, providing the core architecture for polymerase access and genome packaging [29]. M protein underlies the viral envelope (Figure 1B) and orchestrates virion assembly, morphology, and budding by linking the internal RNP to surface glycoprotein arrays. Although complete high-resolution structures of full-length M are limited, recent cryo-ET and subtomogram averaging studies have visualized M as a membrane-proximal lattice that spatially couples to surface F trimers and organizes RNP positioning during budding [26,31]. Functionally coupled to the RNP, the L protein serves as the multifunctional RNA-dependent RNA polymerase (RdRp) of RSV and acts together with the P protein, which functions as a flexible oligomeric cofactor bridging L to the N–RNA template and coordinating multiple steps of RNA synthesis. Cryo-EM structures of the polymerase complex show how P docks onto L and positions the enzymatic machinery relative to the RNP (Figure 1C) [30,47]. These structures provide a foundation for the design of polymerase inhibitors and for mechanistic studies of RNA synthesis.

The M2 gene encodes two regulatory proteins with distinct roles. M2-1 is a transcription antitermination factor that binds RNA and interacts with P to increase polymerase processivity, and its crystal structure has been solved [48]. By contrast, M2-2 modulates the transcription–replication balance, favouring genome replication when expressed, although its structure remains poorly defined [41].

In addition to structural proteins, RSV encodes two non-structural proteins, NS1 and NS2, which suppress host innate immunity by interfering with interferon (IFN) production and signalling. To date, the structures of both NS1 [35] and NS2 [49] have been resolved. Structural and functional studies demonstrate that NS1 forms interfaces that recruit host ubiquitin E3 ligase components to target signalling proteins for degradation, and both NS1 and NS2 disrupt interferon induction and STAT2-dependent signalling pathways. These activities are major determinants of RSV virulence [50].

Together, these primary structures (Figure 1C) define the molecular architecture of RSV and explain how individual viral proteins cooperate to mediate entry, replication, assembly, and immune evasion, providing the foundation for structure-based vaccine and antiviral development.

## 3. RSV Life Cycle

### 3.1. Viral Attachment and Entry

RSV infection is initiated by the G protein, which mediates viral attachment. RSV entry should be viewed as a multistep and still incompletely resolved process.

The viral G protein has been reported to bind to carbohydrates or specific host cell-surface receptors, such as CX3CR1 (CX3C motif chemokine receptor 1) and heparan sulfate proteoglycans (HSPGs) [51], thereby bringing the virus into proximity to the host cell membrane (Figure 1C). At this stage, the F protein remains in a metastable prefusion state, with its hydrophobic fusion peptide sequestered within the interior of the trimer. Upon further contact with the host cell, the pre-F protein has been proposed to first bind to insulin-like growth factor 1 receptor (IGF1R) and subsequently interacts with nucleolin (NCL). The pre-F protein then refolds into a highly stable postfusion configuration (post-F), inserting the fusion peptide into the target membrane and collapsing into a six-helix bundle that draws viral and cellular membranes together. In this framework, IGF1R is best described as a candidate signalling receptor that promotes efficient entry, while NCL is more appropriately viewed as a fusion-associated coreceptor or cofactor recruited to the cell surface during infection [45,52,53]. This process will be discussed in detail in the following sections.

### 3.2. Transcription and Genome Replication

After the genome is released into the cytoplasm, the negative-sense RNA genome encapsidated by the N serves as the template for the viral polymerase [29]. N, L, P, and M2-1 form the RNA synthesis RNP complex [29,32]. Viral transcription and genome replication are catalyzed by the RdRp complex, composed of the L protein and its essential cofactor P protein. Cryo-EM studies of the L-P complex revealed domain organization of L, including the RdRp catalytic core, capping, and methyltransferase modules, thereby illuminating mechanisms of RNA synthesis, capping, and 5′ methylation that are essential for mRNA stability and translation [30,47]. These structures also provide a structural basis for antiviral drug development targeting the polymerase complex. M2-1 functions as a transcription antitermination factor, interacting with P and nascent RNA to enhance polymerase processivity, while M2-2 modulates the switch between transcription and replication [32,42]. RSV transcription is traditionally described as a gradient process, in which gene expression decreases with increasing distance from the 3′ promoter. However, recent studies have shown that this pattern is not universal and can vary depending on viral genotype, with some isolates exhibiting non-gradient transcription profiles [54]. Replication occurs within cytoplasmic inclusion bodies of the host cell [55].

### 3.3. Protein Translation

Viral mRNAs are translated by host ribosomes. Three transmembrane glycoproteins (F, G, and SH) enter the endoplasmic reticulum (ER) and undergo N-/O-glycosylation, folding, and quality control. F protein acquires its prefusion fold in the secretory pathway and is cleaved by furin-like proteases in the trans-Golgi network [41]. G protein is extensively glycosylated and contains mucin-like regions that complicate structural characterization [56]. Proper processing in the ER-Golgi system is required for trafficking of glycoproteins to the plasma membrane, where they become available for assembly into budding virions. In contrast, SH is a small viroporin that oligomerizes within membranes to form ion channel-like assemblies. After processing, F, G, and SH are trafficked to the plasma membrane for incorporation into budding virions.

The remaining viral proteins (N, P, L, M, M2-1, M2-2, NS1, and NS2) are synthesized on free cytosolic ribosomes. N encapsidates genomic RNA to form the RNP complex, while P and L assemble into the RdRp. M2-1 enhances transcriptional processivity, and M2-2 regulates the transcription–replication balance. The M protein accumulates intracellular membranes and later coordinates virion assembly at the plasma membrane. NS1 and NS2 antagonize host innate immune signalling to facilitate viral replication.

### 3.4. Assembly and Budding

At late stages of replication, the M protein is recruited to cytoplasmic inclusion bodies to interact with RNPs, and to lipid rafts to associate with the viral glycoproteins G, F, and SH following their maturation in the ER and Golgi [57,58]. Assembly is orchestrated at membrane microdomains where M protein accumulates beneath the viral envelope and coordinates recruitment of glycoproteins and RNPs [59]. Cryo-ET studies reveal that M forms a lattice coordinating F trimers and RNPs to shape virion architecture, while RNP–M and glycoprotein interactions drive plasma membrane budding and pleomorphic virion formation [26].

## 4. Fusion Machinery

**Figure 2:**
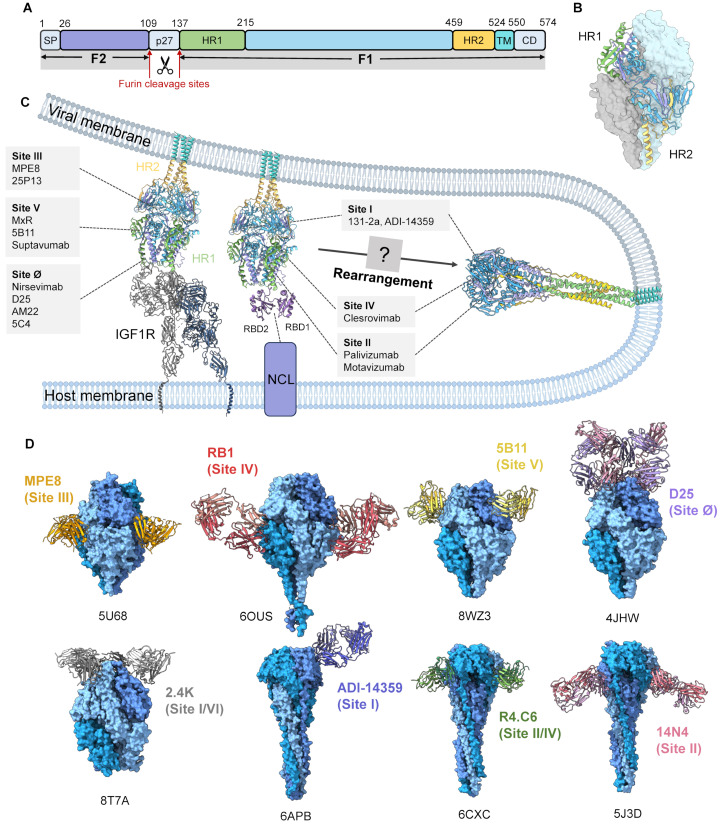
Structure and function of the RSV fusion protein. (**A**) Schematic diagram of the RSV F0 protein domain organization. The F0 precursor is cleaved by the host protease furin into F1, p27, and F2. SP, signal peptide; HR1, heptad repeat 1; HR2, heptad repeat 2; TM, transmembrane domain; CD, cytoplasmic domain. The two furin cleavage sites are indicated. (**B**) The RSV pre-F protein assembles as a trimer (PDB: 4JHW). The distribution of the corresponding domains is shown using the same colour scheme as in (**A**). (**C**) Proposed model of RSV membrane fusion. The pre-F protein on the viral envelope (PDB: 4JHW) initially interacts with IGF1R (PDB: 6JK8), activating intracellular signalling that promotes the translocation of NCL to the host cell surface. The RBD1–2 domains of NCL (PDB: 2KRR) then bind to pre-F, triggering exposure of the fusion peptide and inducing major conformational rearrangements of the F protein, ultimately resulting in its conversion to the postfusion conformation (PDB: 3RRR) and membrane fusion. Major antigenic sites of the F protein and their associated monoclonal and representative antibodies. (**D**) Representative structures of RSV F protein in complex with neutralizing antibodies, highlighting major antigenic sites. Selected examples are shown with corresponding PDB IDs.

The F protein is highly conserved across the two antigenic subtypes, RSV-A and RSV-B, and is the primary target for neutralizing antibodies and structure-based vaccine design [56,60]. Proteolytic activation of the F protein is a prerequisite for membrane fusion, as F is synthesized as an inactive precursor (F0) and cleaved by host furin-like proteases in the secretory pathway to generate the covalently linked F1 and F2 subunits, which forms a pre-fusion trimer on the viral envelope (Figure 2B) [27].

The F protein undergoes complex conformational changes during membrane fusion (Figure 2C). In a proposed entry model supported by airway epithelial and organoid studies, the pre-F protein firstly engages IGF1R on the host cell surface, initiating outside-in signalling. This interaction leads to IGF1R autophosphorylation and activation of the downstream kinase protein kinase Cζ (PKCζ), which in turn drives the trafficking of NCL from intracellular pools, including the nucleus, to discrete sites on the plasma membrane [52]. The recruited cell-surface NCL then acts as a coreceptor. The RNA-binding domains (RBD1–2) of NCL directly bind to the pre-F protein and promote exposure of the fusion peptide and its insertion into the host cell membrane [53]. This process forms an extended intermediate that anchors both the viral and host membranes.

In this model, IGF1R contributes primarily as an entry-associated signalling receptor, whereas NCL functions more as a fusion-associated coreceptor than as a simple attachment receptor. Nevertheless, the precise sequence of host-factor engagement remains less certain.

The F protein then undergoes a largely irreversible refolding process. The two heptad repeat regions, HR1 and HR2 (Figure 2A,B), rearrange to form a long trimeric coiled-coil structure and subsequently fold into a six-helix bundle (6HB) [28]. This folding reaction forcibly draws the viral and host membranes into proximity, overcoming the repulsive forces between lipid bilayers and driving progression from hemifusion to fusion pore formation, ultimately releasing the viral RNP complex into the cytoplasm [41]. Although the prefusion and postfusion states in complex with various neutralizing antibodies are structurally defined (Table 2), transient fusion intermediates remain structurally elusive.

## 5. Vaccine and Antiviral Design

### 5.1. F Protein Structure-Guided Design of Vaccine and Therapeutics

Structural biology has transformed RSV vaccine design by revealing neutralization-sensitive surfaces on the F glycoprotein. More than seven antigenic sites have been identified to date, among which six major sites (Ø, I, II, III, IV, and V) have been well characterized (Figure 2C) [95]. Among these, antigenic site Ø, located at the apex of the pre-F protein, is the most immunologically active and is targeted by many highly potent neutralizing antibodies [96]. Antigenic site V, positioned on a lateral surface of the pre-F trimer, is also recognized by broadly neutralizing antibodies. Early structural studies of the RSV F protein revealed its dramatic conformational transition, providing a mechanistic explanation for the poor efficacy of early vaccine candidates based on post-F [62]. Then, these key epitopes are absent in the postfusion conformation, and immunization with stabilized pre-F antigens elicits substantially higher neutralizing antibody titres and superior protection compared with post-F, establishing prefusion stabilization as the foundation of current RSV vaccine and antibody development strategies [97].

A breakthrough occurred in 2013 with the determination of the pre-F trimer structure in complex with the potent neutralizing antibody D25, which demonstrated that the most effective neutralizing epitopes, including antigenic site Ø, are uniquely exposed in the prefusion conformation [27]. Building on this insight, structure-guided engineering of the F protein led to the development of the DS-Cav1 construct, which stably preserves antigenic site Ø and represents the first rationally designed RSV vaccine antigen [28]. A phase I clinical trial demonstrated a marked increase in neutralizing antibody titres and induction of prefusion-specific antibodies, confirming the clinical feasibility of structure-based RSV vaccine design [60].

In parallel, crystallographic and cryo-EM structures of the F protein in complex with diverse neutralizing antibodies (Figure 2D), including ADI-14359 [64,66], R4.C6 [66], MPE8 [64], and RB1 [72], systematically mapped antigenic sites beyond site Ø, including sites I, II, III, IV, and composite epitopes, generating a detailed epitope atlas that has guided immunofocussing and antigen optimization strategies [98].

More recent cryo-EM studies have further expanded the vaccine design landscape by identifying conserved lateral epitopes on pre-F, including antigenic site V [81] and the newly defined site VI [80]. These epitopes are targeted by broadly neutralizing antibodies and represent promising targets for next-generation vaccine development. In addition, Liang et al. demonstrated that targeting a dynamically flexible region of the RSV F protein can stabilize the prefusion conformation, with cryo-EM confirming a native-like pre-F trimer that elicits protective neutralizing responses comparable to DS-Cav1, thereby introducing a dynamics-guided strategy for next-generation RSV vaccine design [93]. Collectively, these findings transformed RSV vaccine development from empirical antigen selection into a rational, structure-based process and laid the conceptual groundwork for the design and approval of prefusion F-based vaccines, as well as future strategies targeting conserved epitopes and fusion intermediates.

### 5.2. Structure-Guided Antiviral Development Beyond F

While RSV vaccine development has been dominated by prefusion F-based strategies, structural insights into other viral proteins may also inform future vaccine design [18,19]. Emerging evidence suggests that G protein is also being explored in vaccine development, including early-phase clinical studies [99]. Although its full-length structure remains elusive due to heavy glycosylation and intrinsic disorder, high-resolution characterization of its central conserved domain (CCD) has revealed a compact fold harbouring the CX3C motif. This motif acts as a molecular mimic of the host chemokine fractalkine, facilitating critical receptor interactions [44]. This structural clarity has paved the way for the identification of conserved neutralizing epitopes, underpinning the development of G-based immunogens (e.g., ADV110) and therapeutic antibodies designed to elicit broad, cross-strain protection.

Structural elucidations of the SH protein [46], M protein [26], and the virion lattice [31] have primarily informed the fields of viral assembly and antigen presentation. Specifically, the resolution of SH as a pentameric ion channel has redefined it from a functionally obscure accessory protein into a definitive viroporin. M protein organizes a membrane-proximal lattice that coordinates glycoprotein distribution and RNP positioning, providing a structural basis for designing virus-like particle (VLP) and nanoparticle vaccines that better mimic native virion architecture.

At the same time, structural characterization of the RNP and polymerase machinery has had a more direct impact on antiviral development. The N–RNA assembly and L–P complex provide a structural framework for targeting genome packaging, RNA synthesis, and enzymatic activities essential for viral replication, thereby expanding therapeutic strategies beyond entry inhibition [29,30,47]. Structural and mechanistic studies of M2-1 and M2-2 have further clarified how RSV coordinates transcription and replication, highlighting additional regulatory vulnerabilities in viral RNA synthesis [41,42]. In addition, the non-structural proteins NS1 and NS2 have informed live-attenuated vaccine design, since their roles in suppressing interferon signalling and host antiviral responses can be selectively disrupted to reduce virulence while maintaining immunogenicity [35,49,50,100].

Overall, these non-F structural advances demonstrate that RSV structural biology has contributed not only to antigen design but also to the identification of novel therapeutic targets across multiple stages of the viral life cycle. By linking structural features to functional mechanisms, these studies provide a broader framework for the development of next-generation vaccines and antiviral agents that extend beyond F-centred prophylaxis.

### 5.3. Advances in RSV Vaccines

Prophylaxis against RSV relies on three complementary strategies [41,57,101]. Passive immunization with monoclonal antibodies and a limited number of antiviral drugs remain important complementary approaches, although their use is restricted to specific populations and clinical settings [102].

At present, considerable progress has been made in the development of RSV preventive strategies (Table 3), and three vaccines based on stabilized pre-F have been approved: Arexvy, Abrysvo, and mRESVIA. These vaccines elicit robust neutralizing antibody responses by directly targeting highly sensitive epitopes exposed on the pre-F protein [28]. The first RSV vaccine, Arexvy (GSK) [103], is a recombinant RSV vaccine based on a structure-stabilized pre-F protein [104]. It induces high titres of prefusion-specific neutralizing antibodies (RSV PreF3) that inhibit viral fusion. In a phase 3 trial, a single dose significantly reduced RSV-associated lower respiratory tract disease (RSV-LRTD), with efficacy exceeding 80 percent in the first RSV season [105]. Based on these data, the vaccine was approved by the US Food and Drug Administration (FDA) in May 2023 for use in adults aged 60 years and older.

Another bivalent recombinant pre-F protein vaccine is Abrysvo (Pfizer), which is composed of stabilized pre-F proteins from RSV A and B strains, designed to maximize the breadth of neutralizing antibody responses [106]. Clinical trials demonstrated that maternal vaccination during late pregnancy enabled efficient transplacental transfer of RSV-neutralizing antibodies, protecting infants against severe RSV-LRTD during the first six months of life [107]. Accordingly, it is currently the only RSV vaccine approved for maternal immunization to prevent RSV-LRTD in infants from birth to 6 months of age [18]. The vaccine was FDA-approved in May 2023 for adults ≥ 60 years and in August 2023 for maternal immunization, with protection mediated by antibody-dependent inhibition of viral fusion.

In addition to the two vaccines mentioned above, an mRNA-based vaccine, namely mRESVIA (Moderna), was also approved by the US FDA in 2024 [108]. It encodes a structure-stabilized pre-F protein and leverages the same structure-guided antigen design principles established for protein-based pre-F vaccines [109]. Phase 3 clinical trials demonstrated that a single dose significantly reduced RSV-LRTD in adults ≥ 60 years of age by inducing robust prefusion-specific neutralizing antibody responses [110]. The vaccine was approved in 2024 for older adults and confers protection through in situ expression of pre-F, leading to antibody-mediated blockade of viral fusion.

Overall, structure-based targeting of the pre-F protein has defined the current landscape of RSV vaccination. In parallel, multiple companies are pursuing the development of other vaccine formats targeting the F protein, including nanoparticle-based vaccines such as Novavax’s ResVax [111] and VLP vaccines like Icosavax’s IVX-A12 [112].

In addition to prefusion F-based vaccines, other RSV proteins are increasingly being explored as complementary antigenic targets. Among these, the G protein is the most advanced non-F vaccine target. G-based vaccine candidates have entered clinical evaluation: Advaccine’s ADV110 and BARS13 have shown favourable safety, tolerability, and immunogenicity in early-phase studies, supporting the feasibility of extending vaccine design beyond F-only approaches [99,113]. At present, however, G-based vaccines remain clearly less mature than prefusion F-based platforms and should be viewed as complementary rather than replacement strategies.

Some proteins are also being incorporated into multicomponent and live-attenuated vaccine platforms rather than being used only as standalone antigens. Viral-vector candidates such as MVA-BN-RSV were designed to express multiple RSV antigens, including F, G, N, and M2, with the aim of broadening humoral and cellular immune responses [102,114]. This multicomponent design logic is particularly attractive because it may broaden immune coverage beyond highly neutralizing antibody responses alone and potentially strengthen T-cell immunity. In paediatric vaccine development, live-attenuated approaches have exploited non-F proteins indirectly through rational attenuation of NS2, M2-2, and L, thereby reducing virulence while preserving mucosal and cellular immunogenicity [115,116]. In this context, NS1/NS2 and M2-2 are best understood not as dominant standalone antigens but as attenuation determinants that can be engineered to improve the safety–immunogenicity balance of live vaccine platforms.

Representative examples include RSVt (ΔNS2/Δ1313/I1314L), which has advanced to phase 3 evaluation after promising early-phase immunogenicity; MV-012-968, which uses codon deoptimization of G, NS1, and NS2 together with SH deletion; and CodaVax-RSV [117], which incorporates extensive attenuation-related changes including mutations in N, P, M2-1, and L [115,116,118,119]. Taken together, these data indicate that, outside the pre-F paradigm, the most clinically advanced vaccine direction is still G-based antigen design, whereas N-, M-, NS-, and M2-centered approaches are currently more relevant as components of multivalent, vector-based, or live-attenuated strategies than as independent subunit vaccines.

### 5.4. Advances in RSV Therapeutic Agents

To date, three prophylactic monoclonal antibodies have been approved for RSV prevention: palivizumab, nirsevimab, and clesrovimab [120]. Palivizumab was the first monoclonal antibody licensed for RSV prophylaxis and targets antigenic site II on the F protein [121]. It was developed to prevent severe RSV-LRTD in high-risk infants.

Palivizumab neutralizes RSV infection by binding this epitope and sterically inhibiting the conformational rearrangements required for membrane fusion. Structural studies indicate that it does not stabilize the pre-F conformation but instead blocks fusion through steric hindrance [62]. In 2022, nirsevimab [122], a long-acting monoclonal antibody, was approved, addressing the limitation that palivizumab had been the only available prophylactic option for high-risk infants [102]. Nirsevimab is engineered for extended serum half-life and targets antigenic site Ø on the pre-F protein [102]. A single intramuscular dose provides protection throughout an entire RSV season [89]. Large phase 2 and phase 3 clinical trials demonstrated substantial reductions in medically attended RSV lower respiratory tract infection and RSV-associated hospitalization in both healthy and high-risk infants [123]. However, high cost and supply constraints have limited widespread implementation.

More recently, clesrovimab (ENFLONSIA™, MK-1654), a monoclonal antibody developed by Merck, received regulatory approval in the United States in June 2025 for prevention of RSV-LRTD in neonates and infants entering their first RSV season [124]. Unlike palivizumab and nirsevimab, clesrovimab targets a highly conserved epitope at antigenic site IV of the F protein that remains structurally accessible in both prefusion and postfusion conformations. This enables neutralization independent of F-protein conformational state and confers broad activity against RSV A and B subtypes [72].

Beyond passive immunization, ribavirin remains the only antiviral drug historically approved for RSV treatment [125]. Ribavirin is a synthetic guanosine analogue with broad-spectrum activity against RNA viruses, including RSV [126]. It inhibits RSV replication through polymerase interference and lethal mutagenesis mechanisms, a process in which excessive mutation accumulation drives viral population collapse [127,128]. Although aerosolized ribavirin was previously used to treat severe RSV-LRTD, particularly in infants and immunocompromised patients, its routine use in otherwise healthy infants is no longer recommended because of limited clinical efficacy and safety concerns [129,130,131].

Beyond F-targeted prophylaxis, recent structural studies have expanded RSV therapeutic development toward several non-F proteins, particularly the polymerase complex and nucleoprotein. Among these, the L–P polymerase complex is currently the most mature non-F antiviral target. Clinically, both nucleoside and non-nucleoside polymerase inhibitors have advanced into human testing. Lumicitabine showed antiviral activity in challenge studies but failed in hospitalized infants and was associated with dose-dependent neutropenia [132]. More recent candidates, including PC786, EDP-323, JNJ-64417184, obeldesivir, and remdesivir, indicate that polymerase-directed therapy remains the most advanced non-F small-molecule strategy [18,133].

N protein is the second major non-F antiviral target to reach clinical testing. RSV-604, RV-299, and EDP-938 have all entered clinical development, with EDP-938 showing the most encouraging profile, although clinical benefit in naturally infected patients has remained modest so far [134].

By contrast, other protein therapeutic targets remain less mature. The M protein lattice is structurally attractive because of its role in virion assembly and budding, but no M-targeted clinical inhibitor has yet emerged. NS1 and NS2 are also appealing because they suppress interferon signalling and contribute to virulence, yet current efforts remain largely conceptual or preclinical [50]. Therapeutic targeting of G has focused mainly on monoclonal antibodies and immunomodulatory strategies; relative to F-directed antibodies, these programs remain less advanced clinically, although they may additionally modulate RSV-associated inflammation [19,44].

Overall, the non-F therapeutic pipeline is broader than it was only a few years ago, but it remains uneven in maturity. At present, the polymerase complex is the most advanced non-F small-molecule target; N has credible clinical candidates, whereas M, G, NS1, and NS2 remain earlier-stage therapeutic opportunities. Thus, most approved interventions continue to target the F protein, as summarized in Table 3 and Figure 1.

## 6. Future Perspectives

The success of prefusion F-based vaccines has firmly established structure-guided antigen stabilization as the cornerstone of RSV vaccine design, providing a clear structural roadmap for first-generation vaccines and antibody therapeutics. Future studies will likely leverage advanced protein engineering and dynamics-guided design to modulate flexible regions and conformationally coupled sites, with the aim of improving antigen thermostability, expression efficiency, and manufacturability. These improvements are anticipated to extend the durability of immune protection, reduce dependence on cold-chain logistics, and improve global accessibility [70,93,135]. Meanwhile, high-resolution cryo-EM and cryo-ET studies have continued to expand our understanding of the conformational landscape of RSV F, revealing additional conserved neutralizing epitopes as well as transient intermediate conformations during membrane fusion triggering. Together, these discoveries provide a strong structural rationale for broadening vaccine-elicited neutralizing responses and limiting potential immune escape [72,81,85,90].

Nevertheless, key challenges remain, including waning immunity, reduced responsiveness in older adults due to immunosenescence, incomplete definition of correlates of protection beyond serum neutralizing antibodies, and manufacturing and distribution constraints [112,136,137]. Future priorities include affordable immunization strategies for infants and young children; safe orally available direct-acting antivirals; and deeper insight into in situ viral dynamics, including fusion intermediates, F–receptor interactions, and coordinated M–F–RNP conformations during assembly and budding. Achieving these goals will continue to rely on the close integration of cryo-EM/cryo-ET approaches with functional studies. At present, broadly applicable small-molecule antivirals for infants remain unavailable. DS-Cav1 exhibits notable limitations in structural stability and immunogenicity, particularly in the context of aging populations and dependence on cold-chain storage [138]. Further optimization of DS-Cav1 or identification of improved alternatives may enhance stability, immunogenicity, and deployability. Despite advances in prevention, effective therapeutic options remain limited for populations at the highest risk of severe disease [18]. Addressing these challenges will require continued integration of structural biology, virology, immunology, and clinical surveillance to ensure that next-generation RSV vaccines are not only highly effective but also durable, broadly protective, and globally deployable [18,19,102]. Future non-F therapeutic development will likely depend on better translation of structural findings into clinically effective drugs. For polymerase inhibitors, an important priority is to connect inhibitor-binding sites with resistance profiles and clinical performance, particularly given the gap between challenge-model efficacy and results in naturally infected patients [139,140]. For N-directed inhibitors, the key question is whether improved pharmacokinetics and a higher barrier to escape can translate into clearer clinical benefit.

For G, M, NS1, and NS2, current structural studies have already identified biologically meaningful interfaces, but these targets remain underdeveloped therapeutically. In the near term, G may be most relevant for antibody-based or immunomodulatory strategies, whereas M is more likely to inform assembly-targeted antivirals and NS1/NS2 may be more useful in host-directed therapy or rational attenuation design than in direct-acting antiviral monotherapy. More broadly, future progress may require combination strategies that integrate distinct mechanisms of action and reduce the risk of resistance associated with single-target therapy.

## Figures and Tables

**Table 1:** Glossary of RSV proteins.

Abbreviation	Full Name	Functional Category
**NS1**	Non-structural protein 1	Immune antagonist
**NS2**	Non-structural protein 2	Immune antagonist
**N**	Nucleoprotein	RNP component
**P**	Phosphoprotein	Polymerase cofactor
**M**	Matrix protein	Assembly and budding
**SH**	Small hydrophobic protein	Viroporin
**G**	Attachment glycoprotein	Viral attachment
**F**	Fusion glycoprotein	Membrane fusion
**M2-1**	Transcription antitermination factor	Transcription regulation
**M2-2**	Replication regulatory protein	Transcription–replication switch
**L**	Large polymerase protein	RdRp

**Table 2:** Antibody-bound structures of the RSV fusion protein and key structural advances.

Conformation	Binding Molecule		PDB ID	EMDB ID	Ref.
	Name	Type			
**Postfusion**	None		3RKI		[61]
			3RRR		[62]
	Fab 14N4	Neutralizing antibody	5J3D		[63]
	Fab R4.C6		6CXC	EMD-7774	[64]
	131-2a Fab	Non-neutralizing antibody	9HVW	EMD-52444	[65]
	ADI-14359		6APB		[66]
**Prefusion**	Fab D25	Neutralizing antibody	4JHW		[27]
	MPE8		5U68		[67]
	F-VHH-4		5TOJ		[68]
	F-VHH-L66		5TOK		[68]
	Fab 5C4		5W23		[69]
	CR9501		6OE4		[70]
	CR9501		6OE5		
	motavizumab				
	Fab RSD5		6DC3		[71]
	AM22		6DC5		
	RB1		6OUS		[72]
	RSB1		6W52		[73]
	VHH Cl184		7LVW		[74]
	Fab 32.4K and 01.4B		7LUC	EMD-23520	[75]
	ADI-14442		7LUE	EMD-23521	
	AM14		7MMN		[76]
	AM14		7MPG	EMD-23933	
	MxR Fabs		8DG9	EMD-27419	[77]
	AM14 and AM22		7UJA	EMD-26562	[78]
	RSV-199 Fab		8DZW	EMD-27808	[79]
	2.4K Fab		8T7A	EMDB-41089	[80]
	5B11 Fab		8WZ3	EMD-37945	[81]
	5B11 Fab		8WZ5	EMD-37947	
	60 Fab		8ZYM	EMD-60572	[82]
	RSV_2245 and RSV_3301		9MKN	EMD-48331	[83]
	PR306007		9U74	EMD-63931	[84]
	Fab AM22	Neutralizing antibody	6APD		[66]
	ADI-14359	Non-neutralizing antibody			
	JNJ-2408068	Inhibitor	5EA3		[85]
	JNJ-53718678		5KWW		[86]
	lonafarnib		8PHI		[87]
	D25 fab	Neutralizing antibody	8KG5	EMD-37210	[88]
	lonafarnib	inhibitor			
	nirsevimab	Neutralizing antibody	5UDC		[89]
	Fab AM14		4ZYP		[90]
	Motavizumab				
	None	DS-Cav1, laid the structural foundation for prefusion F-based RSV vaccines	4MMU		[28]
		Improved prefusion trimer stability and structural homogeneity over DS-Cav1	5K6F		[91]
		A more stable, conformationally closed prefusion F trimer with more precise presentation of neutralizing epitopes.	8W3E		[92]
		Improved prefusion F stability via dynamics-guided mutations	8YE3	EMD-39188	[93]
		Foldon-free preF; next-generation vaccine design	9B2X	EMD-44117	[94]

**Table 3:** Vaccines (approved) and antivirals on RSV *.

**Type**	**Name**	**Company**	**Antigen**	**Development Phase**	**Population Target**
**Vaccine**	Arexvy (RSVPreF3 OA)	GSK (London, UK)	RSV prefusion F protein (RSVPreF3) + AS01E adjuvant	Approved in 2023	Adults ≥ 60 years
	Abrysvo (RSVpreF)	Pfizer (New York, NY, USA)	Bivalent RSV prefusion F protein (RSV A & B)	Approved in 2023	Adults ≥ 60 years Pregnant women (32–36 weeks gestation) for infant protection
	mRESVIA (mRNA-1345)	Moderna (Cambridge, MA, USA)	mRNA encoding RSV prefusion F protein	Approved in 2024	Adults ≥ 60 years
**Type**	**Name**	**Company**	**Viral Target**	**Development Phase**	**Target Population**
**Monoclonal antibody**	Palivizumab (Synagis)	AstraZeneca (MedImmune) (Gaithersburg, MD, USA)	F protein (site II)	Approved in 1998	High-risk infants and young children
	Nirsevimab (Beyfortus)	AstraZeneca/Sanofi (Cambridge, UK/Paris, France)	F protein (site Ø)	Approved in 2022	All infants entering first RSV season; some up to 24 months
	Clesrovimab (ENFLONSIA™, MK-1654)	Merck (Kenilworth, NJ, USA)	F protein (site IV)	Approved in 2025	Neonates and infants born during or entering first RSV season
	Motavizumab (MEDI-524)	AstraZeneca (MedImmune) (Gaithersburg, MD, USA)	F protein (site II)	Phase III, not approved by US FDA	High-risk infants
	Suptavumab (REGN2222)	Regeneron (Tarrytown, NY, USA)	F protein (site V)	Phase III, failed	Preterm infants
	RSM01	Gates MRI (Cambridge, MA, USA)	F protein (site Ø)	Phase Ia completed	Healthy adults; intended for infant prophylaxis in later development
**Antiviral/** **inhibitors**	Ziresovir (AK0529)	Shanghai Ark Biopharmaceutical (Shanghai, China)	F protein	Phase II, Completed	Hospitalized infants
**Antiviral/** **inhibitors**	Zelicapavir (EDP-938)	Enanta Pharmaceuticals (Watertown, MA, USA)	N protein	Phase IIa	High-risk patients
**siRNA**	Asvasiran	Alnylam (Cambridge, MA, USA)	N protein	Phase IIa/IIb	Lung transplant recipients/immunocompromised adults
	Molnupiravir	Merck (MSD) (Rahway, NJ, USA)	L protein (RdRp)	Phase 2a	Healthy adults
	Remdesivir	Gilead Sciences (Foster City, CA, USA)	L protein (RdRp)	Phase II	Immunocompromised patients
	EDP-323	Enanta Pharmaceuticals (Watertown, MA, USA)	L protein	Phase 2a	Healthy adults
**Antiviral drug**	Ribavirin	Multiple (generic)	Viral RNA synthesis (nucleoside analogue; non-specific)	Approved (not recommended)	Severe RSV infection (historical use, mainly children)

* The literature and regulatory developments were assessed up to 12 March 2026, and regulatory status is reported primarily for the United States, unless otherwise specified.

## Data Availability

No new data were created or analyzed in this study. Data sharing is not applicable to this article.
